# Paths towards efficiency in healthcare

**DOI:** 10.1590/S1516-31802008000600001

**Published:** 2008-11-06

A systematic review to be published in the São Paulo Medical Journal/Evidence for Health Care in January 2009, prepared by teams at the Brazilian Cochrane Center and the Department of Ophthalmology of Universidade Federal de São Paulo (Unifesp), shows that the use of bevacizumab in intraocular injections increases the efficacy of the current treatment for macular degeneration. In relation to photodynamic therapy, the costs due to medications fall by around 99%.

Taking the prevalence data from the United States,^1^ we estimate that there are at least five million cases in Brazil. Thus, with the decrease in costs from around 10,000 dollars per patient to 33 dollars per patient with the new treatment, the potential budgetary impact could be a reduction from 50 billion to 175 million dollars in Brazil alone!

This is just one of the examples that serve to explain several things: the first is that, even with expenditure of two trillion dollars per year, the American healthcare system is unsustainable and leaves millions of people without healthcare coverage. For further information, watch Michael Moore's film "Sicko", which is a must for the medical profession. Secondly, it explains why medical fees in Brazil are getting smaller all the time, since there is always a great chance that the funds will go into new technologies, although in most cases, the effectiveness of such technologies remains unconfirmed by good-quality evidence.

A large proportion of the maintenance of quality and extensive inclusion of the population within the Brazilian healthcare systems, both public and private, is achieved at the cost of reduced payments to healthcare professionals. However, as we have been saying here for many years, there is a new path that can be taken: that of the search for efficacy, effectiveness, efficiency and safety, based on clinical research that is adequately designed and conducted in order to constitute good evidence.

Designing such clinical research is not a great problem, although only a small number of people interested in clinical research in fact have the preparation to do this. The greater difficulty lies in obtaining finance from funding agencies to conduct such studies. In our view, they lack a body of reviewers who are sufficiently technically prepared for this and, with honorable exceptions, their reports are often a muddle between absence of knowledge and the verge of utter cynicism. Just to cite two of them: one classified the Cochrane reviews as unrelated to postgraduate activities (as if mapping out present knowledge to establish its relevance and better define new research did not form part of the search for new knowledge!) and the other took the view that those who publish a lot must have a lot of funds and so would not need financial support! Despite such problems, the number of papers published from Brazilian studies has increased tremendously; in other words, while we have improved our scientific production, there is still a lack of knowledge about how to apply this.

It is worth bearing in mind that the Medical Research Council (MRC) of the United Kingdom considers it unethical to carry out redundant research on matters for which answers already exist, or researches that are outside of the context, or for which the methodology produces unreliable results. The MRC requires systematic reviews to map out the current knowledge that was acquired with adequate methodology, so that research projects can then be designed with questions posed and outcomes defined in such a way as to diminish the doubts regarding the subject. These doubts generally relate to the efficacy, effectiveness, efficiency and safety of the approaches that will be taken sooner or later, to the benefit of science and medical practice.^2,3^

It is no coincidence that the British Medical Journal (BMJ) considers that evidence-based medicine (EBM) is one of the 15 best ideas within medicine to have arisen since 1840. The Cochrane Library is indexed in the Institute for Scientific Information (ISI) and has an impact factor of 4.68. It is the 14^th^ most cited source within clinical medicine. The São Paulo Medical Journal/Evidence for Health Care was invited to become a member of the Web of Science (ISI) because of its number of citations and is now being monitored more closely by that organization.

The search for evidence to assess new health technology requires not only the availability of expertise but also impartiality. For this, it is fundamental that there should be funding agencies with sufficient preparation and impartiality to play a supporting role. In this way, the major conflicts of interest that occur naturally when parties interested in creating, producing and selling fund clinical research into their own products can be avoided. Even when carried out with the greatest rigor, industry-funded research does not attain the necessary credibility, because of the suspicion of conflicts of interest. Fortunately, in Brazil, the Ministry of Health has been taking an increasingly determined and authoritative role in this respect, as has also been occurring in the United Kingdom, Canada, Australia and the United States, through their research quality and health policy agencies.

We take the view that it is also time for there to be a major research agency of worldwide nature, which would bring together funds for an international consortium for assessments that are impartial and independent of conflicts of interest. This would have the purpose of assessing new technologies using funds from producers and the governments of different countries, in order to study areas of human interest and, in particular, to meet the needs of the poorer countries, whose important issues are systematically neglected.

An article prepared following a symposium of American health economists and published by professors at the Wharton Business School, in 2003,^4^ has already predicted economic chaos in healthcare before 2011. It suggested that preventive medicine and the principles of EBM should be applied as a the strategy for economic and healthcare survival, in order to face up to costs that, at that time, were projected to reach 2.7 billion dollars in 2011, in the United States.

Two articles published recently in the Journal of the American Medical Association (JAMA), the medical journal currently with the highest impact factor, in May^5^ and October^6^ 2008, go along the same lines. The article "The ‘3T's’ road map to transform US health care", by Dougherty and Conway,^5^ discusses the way forward to reach a new situation for American healthcare, namely:

- First, the process of innovation needs to be accelerated, i.e. the efficiency of new health technology assessments needs to be increased, instead of having to wait for them at the snail's pace of today. For this, phase I, II and III clinical trials need to be performed rapidly and efficiently;
- Second, evidence-based decision-making needs to be implemented, with regard to both the prevention and the diagnosis and therapy of diseases (topics that can be presumed to have some relationship with science);
- Third, the impact of the new (and old) technologies applied needs to be continuously measured (to obtain evidence).

If anyone has any doubts about the ways to face up to the present world crises in the economy and in healthcare, it seems that opening one's eyes impartially is already a good start. It is good to note that the medical schools of York, in the United Kingdom, and McMaster, in Canada, have managed in 40 years, through innovations in teaching, learning and EBM, to largely reach the level of Oxford and Cambridge, with their approximately 800 years of history.

It should be noted that the one or two trillion dollars with which it is planned to face up to the present world economic crisis could have been saved over the last two or three years, if they had not been wasted on approaches without scientific proof of their effectiveness and safety within many fields: not just within medicine but within all human activities. We hope that this current economic crisis will enable science and technology to make this the century of efficiency, safety and quality of life, within a healthy environment. More lives will be saved, less suffering (morbidity) will occur and the gain in human health will be detectable in terms of better quality of life and social behavior. Since global warming is associated with waste of resources, even nature will benefit! The treatment for macular degeneration may preserve or improve visual acuity, but only learning the use of reason will improve the decision-making process.

Education relating to the principles of EBM for everyone is the inexorable path to follow, for ordinary citizens, health professionals, lawyers, journalists, health economists, teachers and politicians. The path may be stony, but even *Paramecium sp* has a plan B when faced with obstacles. It should, incidentally, be noted that having a plan B is one of the signs used to demonstrate intelligence in the animal kingdom. It is time to accept a change in direction. Could it be that *Paramecium sp* is intelligent? It seems to have a plan B.

## References

[1] Lee PP, Feldman ZW, Ostermann J, Brown DS, Sloan FA (2003). Longitudinal prevalence of major eye diseases. Arch Ophthalmol.

[2] National Institute for Health Research NIHR Health Technology Assessment programme. HTA clinical evaluation and trials: an open call. Guidance for applicants.

[3] Department of Health (2005). Publications policy and guidance. Research governance framework for health and social care.

[4] Knowledge Wharton Health Economics. Code blue: combating rising healthcare costs calls for strong medicine.

[5] Dougherty D, Conway PH (2008). The "3T's" road map to transform US health care: the "how" of high-quality care. JAMA.

[6] Montori VM, Guyatt GH (2008). Progress in evidence-based medicine. JAMA.

